# Data on nucleoid-associated proteins isolated from *Mycoplasma gallisepticum* after intracellular infection

**DOI:** 10.1016/j.dib.2021.107289

**Published:** 2021-08-14

**Authors:** A.I. Zubov, V.G. Ladygina, S.I. Kovalchuk, R.H. Ziganshin, M.A. Galyamina, O.V. Pobeguts, G.Y. Fisunov

**Affiliations:** a Federal Research and Clinical Center of Physical-Chemical Medicine of Federal Medical Biological Agency, Moscow, Russian Federation; b Shemyakin-Ovchinnikov Institute of Bioorganic Chemistry, Moscow, Russian Federation

**Keywords:** *Mycoplasma gallisepticum*, Intracellular infection, LC-MS/MS, Shotgun proteomics, Nucleoid-associated proteins

## Abstract

*Mycoplasma gallisepticum (M. gallisepticum)* belongs to the class of Mollicutes. It causes chronic respiratory disease in avian species. It is characterized by lack of cell wall and reduced genome size. As a result of genome reduction, *M. gallisepticum* has a limited variety of DNA-binding proteins (DBP) and transcription factors. Consequently, the diversity of DNA-binding proteins and transcription factors (TF) in *M. gallisepticum* is limited in comparison with related bacteria such as *Bacillus subtilis*. Studies have shown, however, that mycoplasmas demonstrate a wide range of differential expression of genes in response to various stress factors, which promotes effective adaptation to unfavorable conditions. We assume that in the case of mycoplasmas, which are characterized by a combination of the reduction of known gene expression regulation systems and a high adaptive potential, the coordination of gene expression can be provided due to local changes in the structure and spatial organization of the chromosome. The study of the dynamic changes of the proteomic profile of *M. gallisepticum* nucleoid may assist in revealing its mechanisms of functioning, regulation of chromosome organization and stress adaptation including its changes upon invasion of the host cells.

## Specifications Table

**Table:** 

Subject	Biology
Specific subject area	Proteomics
Type of data	LC-MS/MS data, identification and LFQ data
How data were acquired	DDA LC-MS/MS analysis on a Q Exactive Plus Orbitrap MS (Thermo Fisher Scientific)
Data format	Raw and analyzed data
Parameters for data collection	Shotgun proteomes of nucleoids of *M. gallisepticum* S6 isolated by centrifugation in sucrose density gradient from bacteria in exponential growth phase with synchronous cell division after acute and chronic infection of HD3 cells
Description of data collection	Data were obtained by mass spectrometric DDA analysis of nucleoid-containing fractions with iRT peptides. For each sample type 3 technical sample preparation replicates were analyzed, 18 runs in total.
Data source location	Research and Clinical Center of Physical-Chemical Medicine, Moscow, Russian Federation
Data accessibility	Data were deposited to the PRIDE repository: https://www.ebi.ac.uk/pride/archive/projects/PXD025107 Project accession: PXD025107

## Value of the Data

• This dataset contains detailed proteomic profiles of *M. gallisepticum* nucleoids obtained from cell culture with synchronized cell division after intracellular infection.
• This dataset can be used in research to investigate new nucleoid-associated proteins in genome-reduced organisms.
• The data are valuable for researchers interested in *M. gallisepticum* proteomics.

## 1 Data Description

**Fig. 1:**
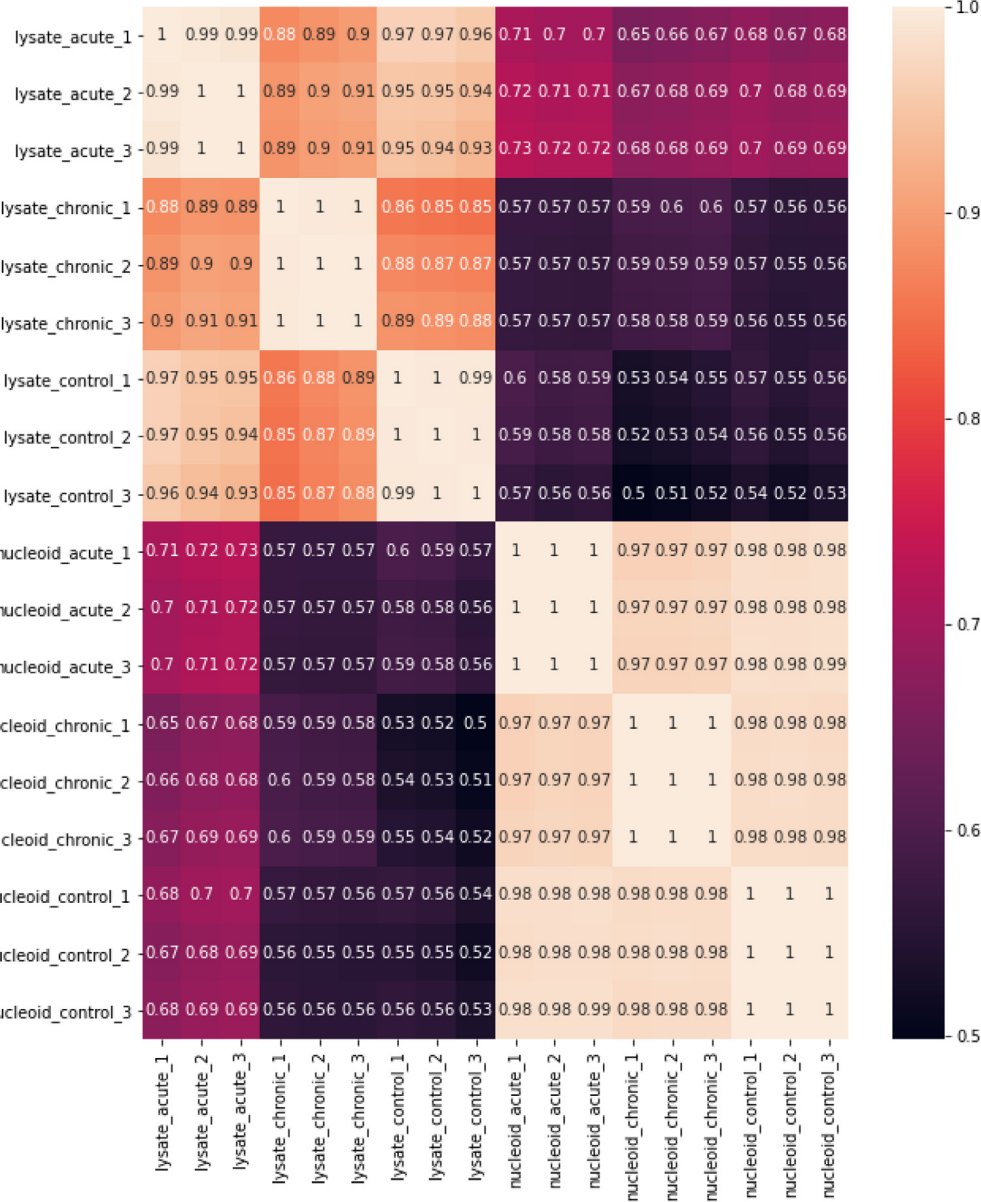
Correlation matrix across all proteomic experiments.

**Fig. 2:**
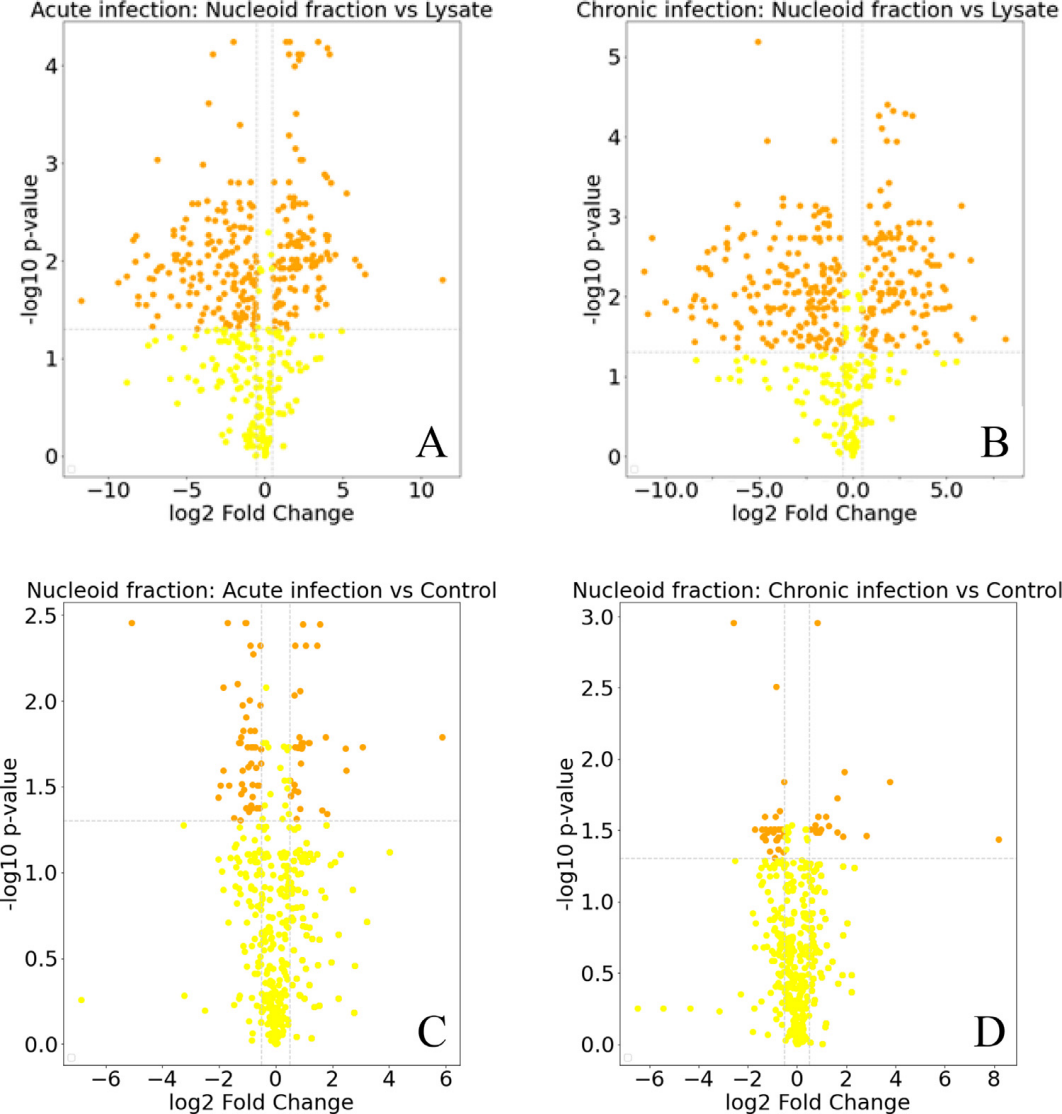
Volcano plots of the resulting quantitative proteomic data. Differential proteins (abs(Log2FC) > 1, adjusted *p*-value < 0.05) are shown as orange dots.

Lately more and more evidence has appeared that one of the mechanisms of regulation of gene expression in bacteria can be local rearrangements of the structure and spatial organization of the chromosome [1,2]. This is most important for mycoplasmas because a small set of transcription factors cannot fully capture the regulation of gene expression. To understand the significance of the structural organization of the chromosome in the processes of regulation of genes and adaptation to stress, it is necessary to identify new TF and DBPs that can take part in dynamic changes in the structure of the chromosome. We previously demonstrated phase transition of the *M. gallisepticum* upon invasion of eukaryotic cells. Proteomic changes affected a broad range of processes including metabolism, translation and oxidative stress response [3]. To search for new potential DBPs that may play a role in the infection process, we isolated nucleoids from *M. gallisepticum* cells before and after infection of the HD3 chicken erythroblast cell line and performed a comparative proteomic analysis of these nucleoids and their corresponding cell lysates. The list of samples is presented in Table 1. LC-MS/MS analysis was carried out on an Ultimate 3000 RSLC nano HPLC system connected to a QExactive Plus mass spectrometer (Thermo Fisher Scientific, USA) [4]. Identification and label-free quantification analysis were performed with MaxQuant 1.6.10.43 software with default settings. The data was searched against *M. gallisepticum* S6 NCBI database partner repository with the dataset identifier PXD025107. (https://www.ebi.ac.uk/pride/archive/projects/PXD025107). Further calculations and visualizations were made in Python 3.7.10. The resulting table of label-free quantification values from MaxQuant, calculated log2 fold change values for each group and raw and adjusted p-values (BH correction), along with Uniport protein and gene names and gene ontology information is presented in the supplementary file S1. The resulting Pearson correlation matrix (Table S1, LFQ values) show good reproducibility between biological replicas (Fig. 1). Volcano plots (Fig. 2, Table S1, LFQ and corrected *p* values) show the number of DNA-binding proteins that change their representation in the nucleoid relative to the cell lysate for acute (Fig. 2A) and chronic infection (Fig. 2B). Lists of proteins enriched (Log2FC > 1, adjusted p-value < 0.05) in nucleoid fractions relative to cell lysate for acute and chronic infections are presented in Table S2 (A-B). The range of protein enriched in *M. gallisepticum* nucleoids before and after acute and chronic infection of HD3 cells is shown in Fig. 2C and D. Table S3 lists differential proteins, the level of which changes by 2 or more times in the nucleoides isolated from cells of *M. gallisepticum* after acute (Table S3, C) and chronic (Table S3, D) infection relative to control nucleoids (before infection).

**Table 1:** List of samples.

#	File	Sample	Case
1	Pobeguts_dif_208_20210304_KS_OP244_nucl-K_1.raw	OP244	nucleoid, control
2	Pobeguts_dif_209_20210304_KS_OP245_nucl-K_2.raw	OP245	nucleoid, control
3	Pobeguts_dif_210_20210304_KS_OP246_nucl-K_3.raw	OP246	nucleoid, control
4	Pobeguts_dif_213_20210304_KS_OP247_nucl-Chr-Inf_1.raw	OP247	nucloid, chronic infection
5	Pobeguts_dif_214_20210304_KS_OP248_nucl-Chr-Inf_2.raw	OP248	nucloid, chronic infection
6	Pobeguts_dif_215_20210304_KS_OP249_nucl-Chr-Inf_3.raw	OP249	nucloid, chronic infection
7	Pobeguts_dif_218_20210304_KS_OP250_nucl-24h_1.raw	OP250	nucleoid, acute infection
8	Pobeguts_dif_219_20210304_KS_OP251_nucl-24h_2.raw	OP251	nucleoid, acute infection
9	Pobeguts_dif_220_20210304_KS_OP252_nucl-24h_3.raw	OP252	nucleoid, acute infection
10	Pobeguts_dif_228_20210305_KS_OP253_lysate_K_1.raw	OP253	lysate, control
11	Pobeguts_dif_229_20210305_KS_OP254_lysate_K_2.raw	OP254	lysate, control
12	Pobeguts_dif_230_20210305_KS_OP255_lysate_K_3.raw	OP255	lysate, control
13	Pobeguts_dif_233_20210305_KS_OP256_lysate_ChrInf_1.raw	OP256	lysate, chronic infection
14	Pobeguts_dif_234_20210305_KS_OP257_lysate_ChrInf_2.raw	OP257	lysate, chronic infection
15	Pobeguts_dif_235_20210305_KS_OP258_lysate_ChrInf_3.raw	OP258	lysate, chronic infection
16	Pobeguts_dif_238_20210305_KS_OP259_lysate_24h_1.raw	OP259	lysate, acute infection
17	Pobeguts_dif_239_20210305_KS_OP260_lysate_24h_2.raw	OP260	lysate, acute infection
18	Pobeguts_dif_240_20210305_KS_OP261_lysate_24h_3.raw	OP261	lysate, acute infection

## 2 Experimental Design, Materials and Methods

### 2.1 Bacterial strains, HD3 cells infection and cell culture synchronization

*M. gallisepticum* S6 was cultivated in a medium containing tryptose 20 g/l, Tris 3 g/l, NaCl 5 g/l, KCl 5 g/l, yeast extract (10%, Helicon, Russia), horse serum (10%, Biolot, Russia), glucose 1% (Sigma) and penicillin (Sintez, Russia) with a final concentration 500 units/ml (BHI medium) at pH 7.4 and 37 °C. Chicken erythroblast cell line HD3 (clone A6 of line LSCC51,52) was obtained from Professor S. V. Razin (Institute of Gene Biology, Russian Academy of Sciences) and was cultivated as described previously [5]. The gentamicin invasion assay was carried out as previously described [6]. We used the concentration of gentamicin 600 µg/ml. Cell lines were infected with the *M. gallisepticum* S6 in a ratio of 1:1000 and cultured for 24 h (acute infection) or 4 weeks (chronic infection) at 37°C and 5% CO_2_. After treatment with gentamicin, HD3 cells were removed from the plate, pelleted by centrifugation at 300 g for 5 min, and diluted in 1 ml of BHI medium. The resulting sample was serially diluted and plated on a semi-liquid medium. The colonies were used to obtain a culture of *M. gallisepticum* to isolate the nucleoid fraction. We used a culture of *M. gallisepticum* with synchronized division. For this, 1% of post-infectious cells were starved for 9 h in a depleted BHI medium (without serum, yeast extract, and glucose) in aerobic conditions at pH 7.4 and 37 °C. After that 10% yeast extract, 20% horse serum and 1% glucose were added. The culture was grown further at 37 °C to the logarithmic growth phase.

### 2.2 Nucleoid isolation and sample preparation for proteomic analysis

Nucleoid isolation was performed as described in [7]. 20 µl of 10% sodium deoxycholate and 2 µl of nuclease mix (GE HealthCare) were added to the nucleoid fraction and incubated for 1 h at 4°C. After incubation, 80 µL of 100 mM Tris – HCl, pH 8.5 with protease inhibitor cocktail (GE HealthCare) was added to the sample. Protein concentration was estimated by BCA Assay (Sigma). Aliquots containing 300 µg of protein material were diluted to 1 µg/µL with 100 mM Tris-HCl, pH 8.5, and tris (2-carboxyethyl) phosphine hydrochloride (TCEP, Sigma) and chloroacetamide (CAA, Sigma) were added to the final concentrations of 10 and 30 mM, respectively. Cys-reduction and alkylation were achieved by 10 min heating of the sample at 85°C. Trypsin (Promega, USA) was added at a ratio of 1:100 w/w to protein amount and incubated at 37°C overnight. Then the second trypsin portion 1:100 w/w was added, and the sample was incubated for 4 h at 37°C. Proteolysis was stopped by adding trifluoroacetic acid to 1%. Precipitated CDNa was removed by ethyl acetate [8]. Samples were subsequently purified on OASIS columns (Waters).

### 2.3 DDA LC-MS/MS analysis

LC-MS/MS analysis was carried out as described previously [7].

### 2.4 Data processing

Identification and label-free quantification analysis were performed with MaxQuant 1.6.10.43 software with default settings. The data was searched against *M. gallisepticum* S6 NCBI database partner repository with the dataset identifier PXD025107. (https://www.ebi.ac.uk/pride/archive/projects/PXD025107). Further calculations and visualizations were made in Python 3.7.10.

## Ethics Statement

This article does not contain any studies involving animals or human participants performed by any of the authors.

## CRediT Author Statement

**A.I. Zubov:** Visualization, Writing – review & editing, Conceptualization, Methodology, Writing – original draft, Data curation; **V.G. Ladygina:** Methodology, Visualization, Writing – review & editing; **S.I. Kovalchuk:** Visualization, Writing – review & editing, Formal analysis; **R.H. Ziganshin:** Visualization, Writing – review & editing, Formal analysis; **M.A. Galyamina:** Visualization, Writing – review & editing, Conceptualization, Methodology; **O.V. Pobeguts:** Visualization, Writing – review & editing, Conceptualization, Methodology; **G. Yu. Fisunov:** Visualization, Writing – review & editing, Conceptualization, Methodology.

## Declaration of Competing Interest

The authors declare that they have no known competing financial interests or personal relationships that might affect the work described in this article.

## Appendix Supplementary materials

Image, application 1

Image, application 2

Image, application 3
